# Research on Curing Water Demand of Cementing Material System Based on Hydration Characteristics

**DOI:** 10.3390/ma14227098

**Published:** 2021-11-22

**Authors:** Wang Yao, Baolin Guo, Zhenyu Yang, Xingxing Yang, Yongzhi Guo, Fangli Zhao, Baomin Wang

**Affiliations:** 1 Shandong Hi-Speed Company Limited, Jinan 250014, China; yaowang4001@sina.com (W.Y.); wu531778564@163.com (Z.Y.); 2 Shandong Province Bridge and Tunnel Engineering Maintenance Technology and New Material Industry Research and Development Center, Jinan 250100, China; 3 School of Civil Engineering, Dalian University of Technology, Dalian 116000, China; guobaolin@sdjtky.cn (B.G.); 15535125675@mail.dlut.edu.cn (X.Y.); 4 Transportation Research Institute of Shandong Province, Jinan 250100, China; Guoyongzhi@sdjtky.cn; 5 Shandong Provincial Key Laboratory of In-Service Bridge Performance Evaluation and Improvement Industry, Jinan 250100, China; 6 Shandong Provincial Concrete Materials and Bridge Structure Engineering Technology Research Center, Jinan 250100, China

**Keywords:** curing water demand, water-binder ratio, mineral admixture, hydration degree

## Abstract

The performance of cover concrete is acknowledged as a major factor governing the degradation of concrete structures. Curing plays a vital role in the development of concrete durability. The effects of different water-binder ratios and mineral admixtures on the curing water demand of concrete were studied by the surface water absorption test. Combined with the characteristics of the hydration heat and chemically bound water of the composition cementing material system, the law of variation for curing water demand was analyzed. The results show that the addition of mineral admixtures can reduce the early hydration rate and hydration exothermic characteristics, and the hydration degree decreases with the increase of mineral admixtures. Due to the filling effect and active effect, the addition of fly ash (FA) and ground granulated blast slag (GGBS) reduces the curing water demand. The curing water demand of cover concrete decreases with the increase of mineral admixture content, and the curing water demand of pure water is the maximum and that of mix FA and GGBS is the minimum. Moreover, there is a strong correlation between the cumulative curing water demand and the chemically bound water content, indicating that the power of water migration mainly comes from the hydration activity of the cementing material system. The results provide a theoretical basis for the fine control of a concrete curing system.

## 1. Introduction

With the continuous development of modern construction technology, the design strength grade of concrete is also constantly improving, especially in terms of the early increase in strength grade [1]. However, in the push for strength, the phenomenon of premature deterioration in the concrete structures has been common [2,3]. Scholars have conducted a large number of scientific research and engineering case analyses [4], and the results obtained are as follows: by mixing mineral admixtures, the cement dosage, hydration heat and volume shrinkage deformation of concrete can be reduced, which refines the internal microstructure of the matrix and enhances the density and impermeability to improve the durability of the structure [5,6]. Low water–cement ratios are often required for the adoption of a composition cementing material system. With the cement hydration and the secondary hydration of mineral admixtures, the water on the concrete surface will constantly move inward, resulting in the loss of the surface water too rapidly, which if serious will lead to surface quality problems of concrete, such as low hydration degree, increased shrinkage and even cracking [7].

As concrete is affected by boundary effects such as reinforcement and formwork during pouring, a layer of mortar layer is formed on the surface [8,9]. The water-binder ratio and the porosity of mortar layer are larger than those of the interior concrete. When exposed to the prolonged and repeated wind and sun, moisture on the surface of concrete will evaporate quickly, which is prone to shrinkage and cracking. Therefore, it is very critical to improve the quality of concrete surface layer [10,11]. The test results obtained by Chrisp et al. [12,13,14] show that the quality of the concrete surface layer is affected by mix ratio, material properties, molding, vibration and curing conditions, among which curing has the greatest influence on the performance of concrete surface layer. Lura et al. [15,16] increased the surface compactness of concrete through favorable curing measures and blocked the connecting channels between the outside world and the inside of concrete, thus improving the durability of concrete. At present, research ideas on curing are that the permeability has been used to characterize the difficulty of water or gas penetration in the concrete, providing new methods for the evaluation of curing effect [17]. Liu [18,19,20] studied the improvement effect of mineral admixtures on concrete permeability through a chloride ion diffusion coefficient test, and the results of the test show that the permeability improvement can be observed after 56 days or even longer. In view of the above, further studies are needed to evaluate the impact of curing methods on the permeability of concrete mixed with mineral admixtures [21]. The water absorption capacity of concrete is also one of the main factors to reflect the permeability of concrete. Shafiq et al. [22,23] determined that mineral admixtures, water–cement ratio and curing time have important effects on the water absorption of concrete through a capillary water absorption test. R.K. Dhir [24,25] compared the water absorption of the cover concrete at different curing ages, and found that the water absorption of concrete cured for 4 days is reduced by 50% on average compared with that of concrete cured for 1 day, indicating that the water absorption capacity of concrete can directly reflect its permeability.

To sum up, based on the hydration exothermic characteristics of different cementing material systems, the curing water demand of a cementing material system under different water-cement ratio is studied in this paper. Combined with test results on their chemically bound water, the influence of the law of hydration degree on the curing water demand is analyzed, and the range of curing water demand is obtained. It is expected that the research results can provide a theoretical basis for the establishment of a curing water demand management system for mineral admixtures concrete engineering.

## 2. Experimental Sections

### 2.1. Raw Materials

The cement (C) used was P·O 42.5 grade ordinary Portland cement produced by Shan Dong Wanhua Chemical Group Co., Ltd., Yantai, China. The basic physical properties of the cement are shown in Table 1. Fly ash (FA) is class I fly ash, and ground granulated blast slag (GGBS) is grade S95. They are selected as mineral admixtures, and the density and loss on ignition of fly ash and slag are 2.25 g/cm^3^, 2.90 g/cm^3^, 4.14% and 1.61%, respectively, and chemical compositions of binders are shown in Table 2. Fine aggregate is supplied by Xiamen ISO Standard Sand Co., Ltd., Xiamen, China, that complied with ISO 679 standard, and tap water was used for mixing.

### 2.2. Mix Ratio

In this test, two water-binder ratios of 0.35 and 0.45 were selected, and cement, fly ash and slag powder were selected to form a composition cementing material system. Among them, 100% pure cement was the reference group, the single dosages of fly ash and slag powder were 15, 30, 20 and 40%, respectively, the dosages of fly ash and slag powder were 15% + 20% and 30% + 40%, and a total of seven test groups were compared. The fixed binder-sand ratio was 0.5, and the detailed mix ratio and compressive strength are shown in Table 3.

### 2.3. Testing Methods

#### 2.3.1. Hydration Heat Test

In order to characterize the hydration process, the total heat evolution was obtained by a TAM Air eight-channel isothermal calorimeter. According to the mix proportion as shown in Table 3, the cement pastes were prepared, and the water binder of mixtures was fixed at 0.35. The working temperature was set at 20 °C, and the control precision was ±1 °C. Measurements were carried out over 72 h [26].

#### 2.3.2. Water Absorption Test

The test adopts 260 × 360 × 20 mm^3^ mortar sample to simulate the surface of concrete, and studies the influence of curing water demand of different admixtures through a surface water absorption test. The specific test steps refer to ASTM C1585 Measurement of Rate of Absorption of Water by Hydraulic Cement Concretes [27]. We cast the sample according to the mix ratio, and placed it in the standard curing chamber for curing (temperature: 20 ± 2 °C; RH: 98%). Only the sample forming surface was retained for the water absorption test and other non-absorbent surfaces were sealed with wax, the sealed sample was immediately put into the container, and water was added to cover the surface of the sample. The daily weight of the sample was recorded (precision 0.01 g). During weighing, the absorbent surface should be rubbed to a surface dry saturated condition.

#### 2.3.3. Chemically Bound Water Test

To explore the effect of different composition cementing material, water–binder ratio and curing age on the hydration degree of cover concrete, the chemically bound water content of composite cement paste was measured. The chemically bound water content of the pastes with water-binder ratios of 0.35 and 0.45 were measured at the curing ages of 3, 7, 14, 28 days. Specimens were broken into small pieces, and soaked in absolute alcohol to stop the hydration immediately. Chemically bound water can be decomposed only at high temperature. In this test, the specimens were first dried at 105 °C in the oven to remove the moisture adsorbed in capillary pores, then put into a muffle furnace at 1060 °C until a constant weight.

## 3. Results and Discussion

### 3.1. Hydration Characteristics of Cementing Material System

The hydration heat mechanism of composite cementing material becomes very complicated due to the application of high volume mineral admixtures in engineering [28]. Therefore, it is necessary to study the effect of different mineral admixtures and their dosage on hydration heat for curing water demand of a composite cementing material system.

**Figure 1:**
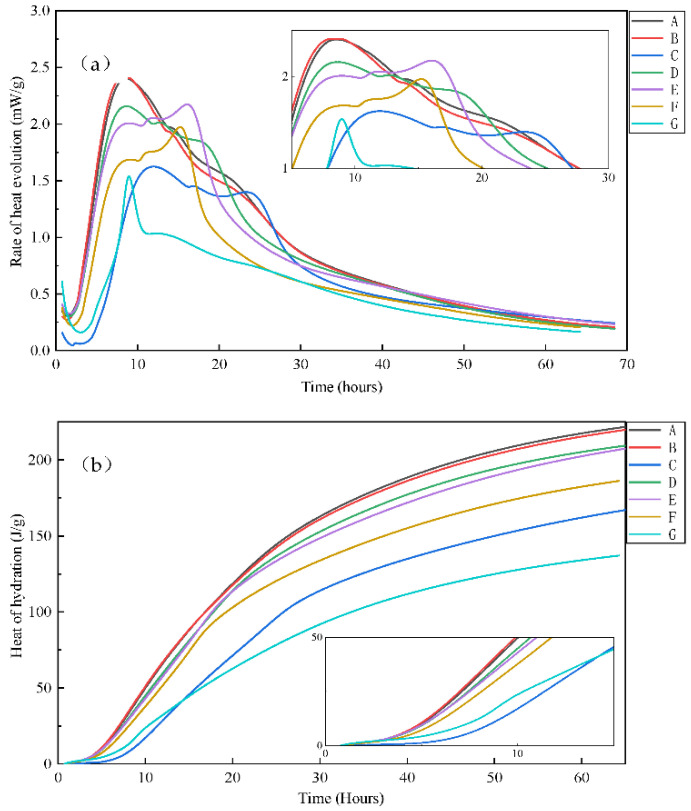
Influence of mineral admixtures on hydration heat. (**a**) Rate of heat evolution; (**b**) cumulative heat of hydration.

The hydration profiles of the different mineral admixtures and dosages were investigated as shown in Figure 1. As can be seen from the figure, the cumulative heat evolved from the blended cements increases with the increasing of time, and heat of hydration is largely concentrated within the first 24 h, after which the hydration exothermic rate gradually decreases. For the pure cement in the reference group, the rate of heat evolved through the hydration of this cement reaches a maximum heat of hydration rate of 2.40 mW/g at 8.5 h, and a second peak of 1.97 mW/g at 13.8 h. The first peak of hydration corresponds to the rapid formation of C–H–S and CH, while the second peak corresponds to the partial AFt transformation to AFm [29].

Compared with curve A, the introduction of mineral admixtures delays the emergence of hydration peak and reduces the rate of heat evolution and cumulative heat of hydration, but that would not alter the peak type of rate of heat evolution. According to curves B and C, when the amount of OPC replaced by FA is 15%, a weak effect on hydration heat evolution is observed. When the amount of FA increases to 30%, the first peak of hydration is delayed to 11.9 h, and the hydration rate decreases by 32.5%. The decrease in heat evolved can be attributed to absorption of Ca^2+^ on the surfaces of fly ash grains with an increase in FA replacement, which will reduce the concentration of Ca^2+^ in the solution, resulting in a delay in nucleation and crystallization of C–S–H and CH. This also could be explained why the concrete blended fly ash has lower strength in the early periods. It can be observed from curves D and E that the rate of heat evolution and cumulative heat of hydration decrease with the replacement of GGBS, but the decrease is smaller than seen in FA. Although both ground granulated blast slag and fly ash belong to active admixtures, GGBS has potential hydraulic properties and higher activity, and GGBS can react directly with water in alkaline environment. The addition of GGBS does not delay the emergence of the first heat release peak, but significantly reduces the rate of heat evolution. The increase of GGBS dosages has a great influence on the second peak of the rate of heat evolution. When the amount of GGBS increases from 20% to 40%, the rate of heat evolution increases from 1.76 mW/g to 2.17 mW/g. The increase of the rate of heat evolution could be accounted for by the superposition of the secondary hydration reaction of slag and the transformation from AFt to AFm in cement, thus leading to a significant increase in the rate of heat evolution [30]. A comparison of curves F and G with curves D and E displays that the total heat released by cement blended FA and GGBS is the minimum. The difference shows that fly ash contributes more to reducing the overall heat release and reaction rate of the composite cementing materials, and GGBS has higher reactivity and hydration heat than fly ash.

### 3.2. The Curing Water Demand of the Cementing Material System

#### 3.2.1. Changes of One-Day Curing Water Demand

**Figure 2:**
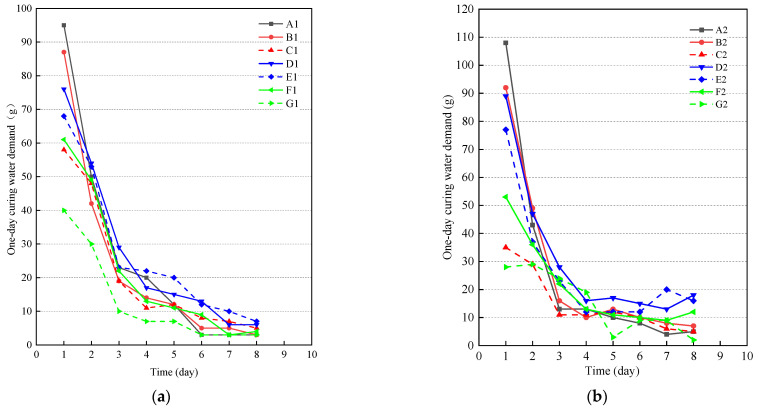
One-day curing water demand of different mineral admixtures. (**a**) 0.35; (**b**) 0.45.

Figure 2 presents the change in 1-day curing water demand of cover concrete with curing age for the different mineral admixtures and dosages with water–binder ratios fixed at 0.35 and 0.45. As can be seen from the law of curing water demand in the figure, in the case of different water–cement ratios and mineral admixtures, the behavior of 1-day curing water demand are similar with the increase of curing age. From the results, it can be observed that the curing age of 1 day has the highest curing water demand. With the increase of curing age, the water absorption rate is fast in the first 3 days, and the curing water demand remains stable after 7 days.

Combined with hydration characteristics of the cementing material system in Section 3.1, it can be seen that on the first day it displays the fastest heat evolution rate and the most hydration products. With the rapid hydration, the samples need to absorb enough water to fill the initial water-filled space. Beyond 1 day, the hydration reaction of the specimens enters the deceleration stage. Comparing the slope of the first 3 days and 7 days, a drastic water absorption rate at 3 days is observed owing primarily to the hydration superimposed effect of the unhydrated cement particles hydration and the secondary hydration reaction of the mineral admixture. After 3 days, the hydration reaction of the specimens enters the stable stage. Hence, the water absorption rate of the sample slows down and the curing water demand tends to be stable accordingly.

It can be seen from the comparison between Figure 2a,b that changes in water-binder ratio and mineral admixtures has a remarkable effect on the one-day curing water demand. At a constant curing age, the one-day curing water demand tends to increase with water-binder ratio from 0.35 to 0.45. Due to an obvious increase in the amount of mixing water, the distance of the cement particles increases compared to 0.35, making the contact area between cement particles and water greater, resulting in the samples with water–binder ratio of 0.45 obtaining a high hydration degree. Therefore, the 1-day water absorption increases with the increase of water–binder ratio. For the first day, the curing water demand of pure cement is the largest. As the hydration activity of the mineral admixture is activated, the 1-day curing water demand of the composition cementing material system is gradually greater than pure cement. Due to the filling effect, the 1-day curing water demand decreases with the increase of admixture dosage. It is worth noting that the 1-day curing water demand of fly ash is more sensitive to the dosage. When the dosage of fly ash increases from 15 to 30%, the water absorption decreases by 32.2–60.8%, which is lower than the 1-day curing water demand of slag. This is because fly ash acts as a physical filler to make cement particles densely packed, which improves the compactness of the initial hardened body structure [31]. Moreover, due to poor activity, few hydration products of fly ash are generated, resulting in slow water consumption and a corresponding decrease in curing water demand, which is consistent with the testing results of a sudden decrease in hydration exothermic.

#### 3.2.2. Changes of Cumulative Curing Water Demand

**Figure 3:**
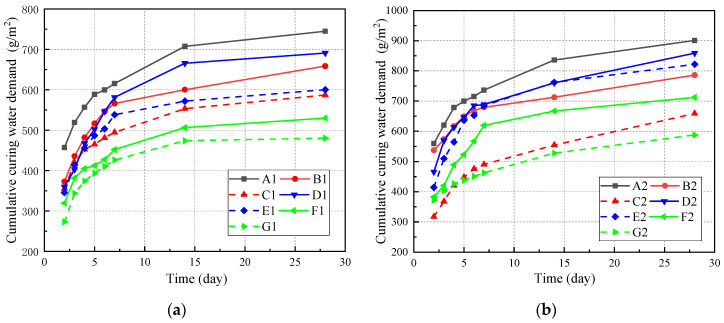
Cumulative curing water demand of different mineral admixtures. (**a**) 0.35; (**b**) 0.45.

Figure 3 presents the change in cumulative curing water demand of cover concrete with curing age for the different mineral admixtures and dosages with water–binder ratios fixed at 0.35 and 0.45. As observed from Figure 3, the specific cumulative curing water demand increases with the increase of the curing age, but the later growth decreases gradually. For the first 7 days of curing, the cumulative curing water demand increases rapidly. Later, the hydration rate slows down, and the cumulative curing water demand increases slowly, and almost stops increasing after 14 days. The cumulative curing water demand of pure cement at 3 days, 7 days and 14 days are 69.6, 82.4 and 95.0% of 28-day cumulative curing water demand, respectively. The test results of Figure 3 also prove that appropriate water curing time is necessary.

It can be seen from the comparison between Figure 3a,b that the water–binder ratio is increasing, and the specific cumulative curing water demand of cover concrete is also growing. At the curing age of 28 days, the cumulative curing water demand of A2 is 901 g/m^2^, which is 1.2 times that of A1. Compared with pure cement, the addition of the fly ash and GGBS can reduce the cumulative curing water demand of the composition cementing material system and with the increase of mineral admixtures dosage, the cumulative curing water demand decreases significantly. When only considering the influence of components in the cementing material system on water absorption without considering environmental factors, the range of cumulative curing water demand for pure cement, fly ash, GGBS and a mixed FA and GGBS cementing material system is 745 ~900 g/m^2^, 660~786 g/m^2^, 691~860 g/m^2^ and 530~712 g/m^2^, respectively. The influence of mineral admixtures on the total water demand is as follows: pure cement > GGBS > fly ash > mix FA and GGBS.

### 3.3. Mechanism Analysis of Curing Water Demand

Concrete surface curing water demand is closely related to the density of microstructure, which depends on the degree of surface hydration. This means that the higher the degree of hydration obtained, the better the compactness of concrete will be. Therefore, the surface of the water absorption test sample was taken as the sample of the chemically bound water test. Based on the hydration characteristics of the cementing material system, the evolution law of curing water demand was analyzed through its hydration degree.

#### 3.3.1. Chemically Bound Water

**Figure 4:**
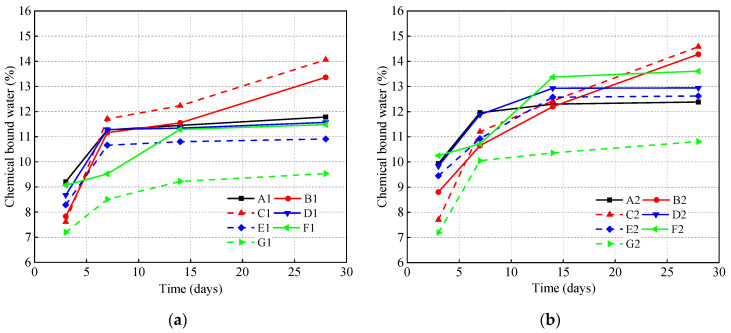
Influence of mineral admixtures on chemically bound water content. (**a**) 0.35; (**b**) 0.45.

To a certain extent, chemically bound water can qualitatively characterize the degree of reaction of the cementing material system. However, the hydration reaction degree of different cementing material systems cannot be directly compared by chemically bound water content due to the difference in the capacity of combination with water. Figure 4 and Table 4 show the variation of the chemically bound water content of different mineral admixtures and dosages with the water–binder ratio fixed at 0.35 and 0.45 as the increase of curing age. It can be observed that the content of chemically bound water increases with the increase of curing time. For the first 3 days of the hydration, the content of bound water increases rapidly; and for the first 7 days of the hydration, the chemically bound water content continues to grow at a relatively fast rate. Seven days later, the content of bound water tends towards stability. At a curing age of 7 days, the content of chemically bound water of pure cement and slag is more than 95% of 28 days, indicating that the hydration of pure cement and GGBS is adequate. The content of 7-day chemically bound water of fly ash is less than 85% that of the 28-day sample, hence, lengthy wet curing is of great importance to the retention of moisture on cover concrete and the hydration of binder. It is worth noting that the content of chemically bound water will continue rising, especially for fly ash.

By comparing Figure 4a,b, it can be seen that water–cement ratio and mineral admixtures have significant effects on chemically bound water. Increasing the water–binder ratio from 0.35 to 0.45 results in the content of chemically bound water increasing, and the increase of the water–cement ratio is conducive to the improvement of hydration degree. That is why the increase of the water–cement ratio results in the enhancement of the curing water demand. For the first 7 days of the hydration, the chemically bound water content of pure cement in the reference group is the highest and tends to be stable gradually. After 7 days, the chemically bound water content of the compound cementing material system will continue to increase, and the 28-day chemically bound water content of fly ash exceeds that of pure cement. The reason for this phenomenon is that the cement hydration reaction dominates in the early stage, and the higher the cement that is contained, the higher the chemically bound water content will be. As the content of CH precipitated in cement hydration increases, the mineral admixtures are in an alkaline medium, which stimulates the secondary hydration of fly ash and the potential hydraulic properties of slag, which is consistent with the conclusion of B.W. Langan et al. [32].

By comparing the curve characteristics of cumulative curing water demand and chemically bound water content, it can be found that both of them show a similar changing rule. With the increase of curing age, the degree of hydration keeps improving, which results in the improvement of the microstructure’s compactness. The reason for the stabilization of growth of the cumulative curing water demand during the later period is the smaller gel pores block the passage of water. An increase in the cumulative curing water demand of the cementing material system is observed for all of them in the order: pure cement > GGBS > fly ash > mix FA and GGBS. As discussed regarding the hydration heat above, the main reason can be seen as: the larger the cement content, the higher the C_3_S and C_3_A, which reacts explosively with water, resulting in numerous hydration products being formed. Furthermore, the absolute volume of the hydration products is smaller than the original volume of cement and water, and the newly formed hydration products cannot occupy the previous water-filled space, leading to negative pressure in the capillary pores. In this case, a continuous migration and supply of water would be needed to balance. Therefore, the more the cement content in the cementing material system, the more the water that will be required. The reactivity of GGBS to hydration is very different to that of fly ash at early ages, and this is due to the contribution of some self-hardening of GGBS. In addition to the glass body, its composition also contains a small amount of dicalcium silicate and mullite crystals, etc., which results in the hydration degree of GGBS being greater than that of fly ash. This is the reason why the curing water demand of GGBS is greater than that of fly ash.

#### 3.3.2. Correlation Analysis between Curing Water Demand and Chemically Bound Water

**Figure 5:**
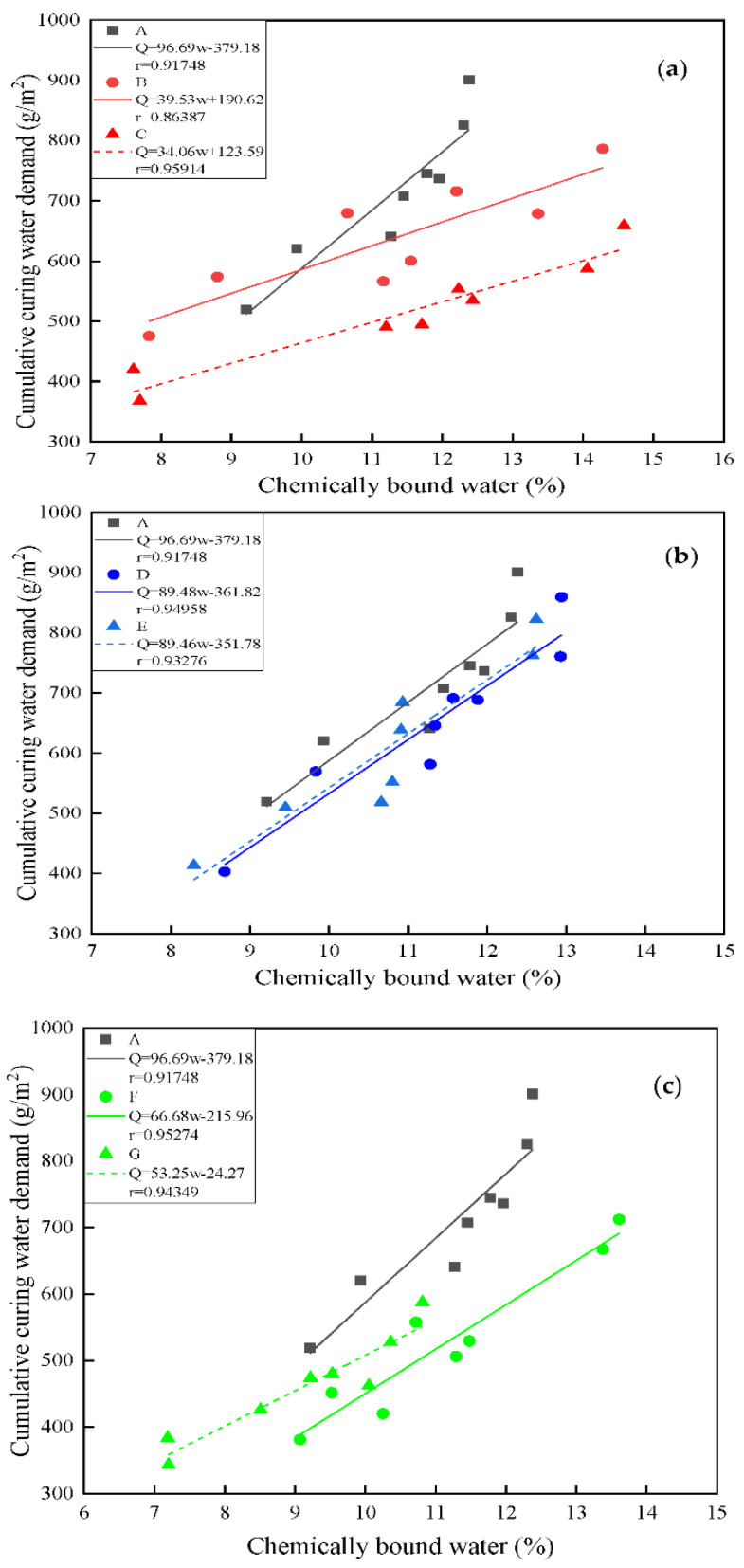
Relationship between cumulative curing water demand and chemically bound water content. (**a**) FA; (**b**) GGBS; (**c**) mixture of FA and GGBS.

On the basis of the above analysis, it is believed that the cumulative curing water demand is closely related to the hydration degree. Therefore, the data of the cumulative curing water demand and the chemically bound water content on 3 days, 7 days, 14 days and 28 days of different cementing material systems are fitted. The fit parameters of the different cementing material systems are given in Figure 5, where Q and w represent cumulative curing water demand and chemically bound water content, respectively, and r is the correlation coefficient of both. These results are consistent with the observed water absorption behaviors, indicating that the degree of hydration has an obvious impact on the curing water demand.

Obviously, Figure 5a–c exhibit the high correlation between cumulative curing water demand and bound water content, as the fit lines of all specimens possess coefficients of determination (R) greater than 0.9. Thus, the cumulative volume of water absorbed by the specimen increases linearly with chemically bound water content. The reason is that when the sample is immersed in water all the time, the driving force of water migration into the sample is mainly the hydration reaction of the cementing material. The slope of the fitting linear equation is defined as the extent of the curing water demand per unit area impact of the unit chemically bound water, and the slope of different cementing systems shows the trend of pure cement > GGBS > fly ash. The law of the slope of the mixed fly ash slag is not obvious, and this difference is believed to be related to the difference in hydration reactivity and the capacity of the water combination of the cement component, resulting in a complicated influence on the curing water demand.

## 4. Conclusions

This study demonstrates that the amount and the kind of mineral admixture can impose on the properties of cement. Its impact on the hydration characteristics and curing water demand of the cementing material system was studied and the mechanism behind this was investigated to gain a more profound understanding of the interaction between hydration degree and water absorption behaviors. The following conclusions can be drawn from the results obtained in this investigation:

1. The curing water demand decreases with the extension of the curing time. The curing water demand of samples is the largest and the water absorption rate is fast during the first 3 days of curing age. At a curing age of 7 days, the curing water demand is basically stable. After 14-day curing, the cumulative curing water demand will not increase.
2. The range of cumulative curing water demand mainly depends on the component and water–cement ratio of the cementing material system. The cumulative curing water demand of samples increases with the increase of the water binder ratio. Among four kinds of cementing material systems, the cumulative curing water demand of pure cement is the maximum (about 900 g/m^2^), then slag, and that of fly ash is the minimum.
3. There is a good correlation between the curing water demand of concrete and the hydration degree of cementing materials. There is an increase of chemically bound water versus time due to curing water consumption by hydration reactions. Hence, appropriate curing water demand is important to improve the hydration degree of cover concrete.

## Figures and Tables

**Table 1:** Physical properties of the cement.

Standard Consistency Water Consumption	Setting Time (min)		Fineness (%)	Soundness	Flexural Strength (MPa)		Compressive Strength (MPa)	
(%)	Initial Setting	Final Setting	80 μm		3 d	28 d	3 d	28 d
28.4	205	260	0.5	Qualified	4.8	6.8	22.6	44.8

**Table 2:** Chemical compositions of the binder (%).

Material	CaO	SiO_2_	Al_2_O_3_	Fe_2_O_3_	K_2_O	SO_3_	Na_2_O	MgO
cement	60.00	23.20	6.90	2.58	0.88	3.97	0.28	1.74
Fly ash	2.71	47.20	37.62	4.55	1.15	2.06	0.49	2.59
GGBS	39.59	33.89	14.22	0.91	0.64	2.96	0.36	6.43

**Table 3:** Mix proportions of the specimens.

Mix Code	W/B	Cement (Kg/m^3^)	Fly Ash (Kg/m^3^)	GGBS (Kg/m^3^)	Sand (Kg/m^3^)	Water (Kg/m^3^)
A1	0.35	2636	0	0	5273	923
B1		2241	395	0	5273	923
C1		1845	791	0	5273	923
D1		2109	0	527	5273	923
E1		1582	0	1054	5273	923
F1		1714	395	527	5273	923
G1		1318	791	527	5273	923
A2	0.45	2636	0	0	5273	1186
B2		2241	395	0	5273	1186
C2		1845	791	0	5273	1186
D2		2109	0	527	5273	1186
E2		1582	0	1054	5273	1186
F2		1714	395	527	5273	1186
G2		1318	791	527	5273	1186

Notations: B, binder (C + FA + GGBS); W, water; S, sand; A, pure water; B and C, fly ash-cement binder; D and E, slag-cement binder; F and G, mix fly ash and slag-cement binder.

**Table 4:** Chemically bound water content.

Mix Code	W/B	3 d	7 d	14 d	28 d	7/28	14/28
A1	0.35	9.21	11.27	11.45	11.78	95.67%	97.20%
B1		7.83	11.16	11.55	13.36	83.53%	86.45%
C1		7.61	11.71	12.23	14.06	83.29%	86.98%
D1		8.68	11.28	11.335	11.57	97.49%	97.97%
E1		8.29	10.66	10.80	10.91	97.71%	98.99%
F1		9.07	9.52	11.285	11.48	82.93%	98.30%
G1		7.2	8.51	9.22	9.53	89.3%	96.75%
A2	0.45	9.93	11.96	12.3	12.38	97.24%	99.35%
B2		8.8	10.65	12.2	14.28	74.58%	85.43%
C2		7.7	11.2	12.43	14.58	76.82%	85.25%
D2		9.83	11.88	12.925	12.94	91.81%	99.88%
E2		9.45	10.93	12.575	12.62	86.61%	99.64%
F2		10.25	10.72	13.375	13.61	78.77%	98.27%
G2		7.19	10.00	10.36	10.81	92.51%	95.84%

## Data Availability

The data presented in this study are available on request from the corresponding author.
